# Using PDE inhibitors to harness the benefits of calorie restriction: lessons from resveratrol

**DOI:** 10.18632/aging.100442

**Published:** 2012-03-04

**Authors:** Jay H. Chung

**Affiliations:** Laboratory of Obesity and Aging Research, Genetics and Developmental Biology Center, National Heart Lung and Blood Institute, National Institutes of Health, Bethesda, MD 20892

Resveratrol is a polyphenolic compound produced in some plants in response to stress, such as injury or infection [1] and is present most notably in red wine. A number of seemingly unrelated health benefits have been attributed to resveratrol, including protection against type 2 diabetes, cancer, heart disease, inflammation and neurodegenerative diseases. However, what these diseases have in common is their association with aging. Therefore, when resveratrol was reported to be a Sirt1 activating compound (STAC) [2], it captured the imagination of the field of aging.

The controversy regarding the mechanism of action for resveratrol arose when a series of papers demonstrated that it activated Sirt1 only if the substrate is attached to a fluorophore or a bulky amino acid [3-7]. However, resveratrol activated Sirt1 in vivo. One potential explanation is that the peptide modifications somehow mimicked the structure of the substrate in vivo. Another potential explanation is that resveratrol indirectly activates Sirt1 by targeting another protein. It has been known for some time that resveratrol indirectly activates AMP-activated protein kinase (AMPK) [8], a well-known regulator of energy metabolism that is also activated by calorie restriction (CR) [9,10]. We and others showed that resveratrol-mediated activation of AMPK increases NAD^+^, the cofactor for Sirt1, as well as Sirt1 activity [11,12]. Consistent with the central role of AMPK in resveratrol action, the metabolic effects of resveratrol disappeared in AMPK knock-out mice [12]. These findings, in conjunction with the observation that resveratrol-mediated activation of AMPK does not require Sirt1 [12], indicated that AMPK is upstream of Sirt1 and that the direct target of resveratrol is upstream of AMPK.

One of the proposed mechanisms by which resveratrol activates AMPK is inhibition of ATP production. However, except at high concentrations of resveratrol (>100 μM), ATP levels do not decrease in the time frame of AMPK activation [13,14], suggesting another mechanism of action. In response to conditions that decrease serum glucose such as CR, glucagon and catecholamines are released. These hormones stimulate adenylate cyclases (AC), resulting in increased cAMP production. To explain the CR-mimetic effects of resveratrol, we measured cAMP levels in resveratrol-treated myotubes and discovered that resveratrol, at low micromolar concentrations (<10 μM), increased cAMP levels [15]. After ruling out the possibility that resveratrol activates AC, we discovered that resveratrol increased cAMP levels by competitively inhibiting a number of cAMP phosphodiesterases (PDEs), which degrade cAMP. We tested PDEs 1-5 and found that resveratrol inhibits PDEs 1, 3 and 4. cAMP, in turn, activates AMPK by increasing the activities of the AMPK kinases CamKKβ and, in some conditions LKB1, via cAMP effector proteins Epac1 (cAMP guaninenucleotide exchange factor) or PKA, respectively. In addition, PKA-mediated phosphory-lation of S434 has been shown to activate Sirt1 [16]. Thus, increasing cAMP levels can activate Sirt1 by a number of pathways.

Since there are 11 PDE family members, each with different properties and tissue expression patterns, it would be impossible to mimic all of resveratrol effects with just one PDE inhibitor. However, PDE4 is the predominant PDE activity in skeletal muscle, the tissue where the metabolic effects of resveratrol are best elucidated. We found that the PDE4 inhibitor rolipram was sufficient to activate AMPK and Sirt1 in myotubes and to reproduce, at least qualitatively, the metabolic effects of resveratrol in skeletal muscle, as well as to improve glucose tolerance in obese mice [15]. It is unlikely that inhibition of PDE4 alone or of cAMP PDEs together explains all of the effects seen with resveratrol. The target(s) of resveratrol will most likely depend on the tissue, the effects of interest and the organism being studied.

One area where we lack understanding is the intracellular concentration of resveratrol. The serum level of unmodified resveratrol is low (submicromolar to low micromolar) because most resveratrol in serum is present in the conjugated form (e.g. glucuronide). However, tissues such as skeletal muscle have glucuronidases, which can potentially removed the conjugate and increase the intracellular levels of unmodified resveratrol far above those in the serum.

The mechanism by which novel chemical entity (NCE) STACs activate Sirt1 in vivo is also under question because like resveratrol, they do not activate Sirt1 against native substrates in vitro, suggesting that they may activate Sirt1 indirectly in vivo [5,7]. Interestingly, analyses of off-target activities of NCE STACs SRT1720, 2183 and 1460 showed that they are stronger PDE inhibitors than resveratrol [7], raising the possibility that they too may be activating Sirt1 in vivo by inhibiting PDEs, at least in part.

In addition to resveratrol, other natural compounds that have been identified as STACs such as butein, fisetin and quercetin have also been identified to be PDE inhibitors [2,17]. This raises the question as to why so many compounds that are identified as STACs using the flurophore-tagged substrate turn out to be PDE inhibitors. We can only speculate at this point, but one possibility is that by coincidence, the structure of the Sirt1 STAC-binding pocket has some similarity to the PDE catalytic pocket.

Whether resveratrol can activate Sirt1 directly in addition to activating it indirectly (via PDE inhibition) remains to be seen. Even if resveratrol can activate Sirt1 directly in vivo, it is not clear how much this effect will add to the well-known anti-inflammatory and antidiabetic effects produced by PDE4 inhibitors alone (e.g. the FDA-approved PDE4 inhibitor roflumilast) [18]. This question may take a while to answer.

In conclusion, the discovery of the resveratrol-PDE link suggests that PDE4 inhibitors, possibly in combination with other PDE inhibitors, may be useful for mimicking CR and for treating aging-related diseases.
